# Dusty Shoes: Appalachia Wisdom Fertilizing the Future of Religious Leadership

**DOI:** 10.13023/jah.0101.05

**Published:** 2019-04-30

**Authors:** Jill Crainshaw

**Affiliations:** Wake Forest University, crainsjy@wfu.edu

**Keywords:** Appalachia, faith, religion, networks

## Abstract

Dust from their journeys through the hills and hollows of Appalachia clings to their shoes and has forever shaped their vocational journeys. This is a refrain I have distilled from the reflections of students who have participated in Wake Forest University School of Divinity’s multicultural contexts course that includes a 10-day sojourn in the mountains of North Carolina.

Dust from their journeys through the hills and hollows of Appalachia clings to their shoes and has forever shaped their vocational journeys. This is a refrain I have distilled from the reflections of students who have participated in Wake Forest University School of Divinity’s multicultural contexts course that includes a 10-day sojourn in the mountains of North Carolina. Funded by a grant through the Appalachian Ministries Educational Resource Center (AMERC) in Berea KY, the course invites students to consider wisdom about life, faith, and the human and ecologic communities that have been birthed over many centuries in the Appalachian region and that continue to flourish and emerge there today.

**Figure:**
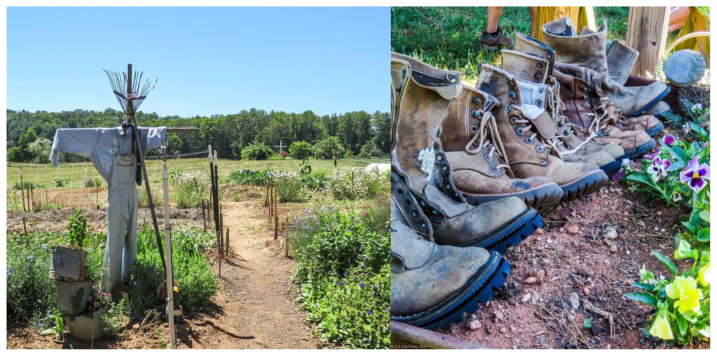


Meredith Doster, a religious scholar from Weaverville NC, taught this year’s course. Titled “I Love to Tell the Story: Appalachia on Our Minds,” the course, in Doster’s words “explored the nation’s longstanding fascination with a region proclaimed as holy Other” (M. Doster; personal communication; March 2019). Called in previous iterations “Fierce Landscapes: Listening to the People or Appalachia,” the course includes site visits to churches, farms, healthcare organizations, public schools, and agencies where students have opportunities for conversations with religious, business, civic, and other regional leaders.

The 2016 version of the course took divinity student Patrick Cardwell (MDiv., ‘16) back to the place where he earned his undergraduate degree, Appalachian State University. “The most memorable part of the trip for me was learning to see the land in a different light,” Cardwell said. “I’ve been fascinated by mountains since my dad first took me to Boone when I was six. The gorgeous views, thrilling overlooks and challenging terrain on a hike are compelling for me.” Course readings and site visits encouraged Cardwell to ask himself what the land of Appalachia is telling us today about faith, community, and care of creation. “One Appalachia farmer phrased it like this,” Cardwell remembers. “‘If the land suffers, we suffer.’” Ecologic well-being and communal well-being are inseparable in Appalachia. (P. Cardwell; personal communication; March 2019.)

This insight reflects what has been at the heart of the course since the School of Divinity first started including it in our curriculum nearly 20 years ago. The health and well-being of human communities are connected to the health and well-being of the geographic places where people live, work, and play. School of Divinity professor Fred Bahnson and his co-author Norman Wirzba put it this way: “Genuine human flourishing depends upon the health of the land and its creatures. God has a different vision for us, a vision in which people live in reconciled rather than exploitive relationships with creation.”^1^

Mr. Bahnson, in consultation with several faculty and staff colleagues, directs the School of Divinity’s “Food, Faith, Health, and Ecological Well-Being Program.” Designed to create networks for faith leaders across North Carolina and beyond, the program includes an annual Summer Institute in Food, Faith, and Ecology that convenes on the campus of Warren Wilson College in Swannanoa NC. A primary aim of the program is to offer educational resources for faith leaders who are working at the intersection of food insecurity, health disparities, and ecologic crises in their ministry contexts. Religious, civic, business and other public leaders in the mountains and valleys in and near Warren Wilson have much wisdom to offer to this vital and challenging work.

At the center of the work our school does in, and connected to, the Appalachia region is a leadership value I highlighted in an essay in *Grounding Education in the Environmental Humanities*: *Exploring Place-Based Pedagogies in the South*.^2^

Effective religious leaders are ministers in place: deeply engaged with the land, history, people and patterns of particular places. They are committed to the health and well-being of their locale and its inhabitants. Through the lens of the particular issues of a place, religious leaders are able to see more vividly the web of connections of the local context with a global environment and global economy. Leadership in place is a practice, not a set of traits or qualities; leadership is worked out through direct, persistent, active engagement with the needs, hopes, and possibilities of a community.^2^

The School of Divinity hopes that through our Appalachia programs religious leaders will become more aware of the possibilities and challenges and the webs of connections rooted and emerging in our North Carolina mountains.

We also hope that students will return to Winston–Salem from their mountain experiences asking the kinds of questions Meredith Doster posed to them at the outset of this year’s course, as they began to peel back layers of myths and assumptions that sometimes hide the beauty and complexity present in the diverse places we call Appalachia:

As we convened our first circle, students were surprised to discover that Wake Forest’s main campus is located in an Appalachian-designated county. Learning that they had woken up in the region before even setting out on the travel course, the students considered what it might mean to locate “Appalachia” in this particular place. What would this pilgrimage into holler-holiness teach us about our own homeplaces? Which sacred stories would we sacrifice along the way? Would we come to know ourselves in the Appalachias of our own making? (M. Doster; personal communication; March 2019)

The roads along the I-40 corridor between Winston-Salem and various towns and rural areas in Appalachia have been well-traveled over the years by Wake Forest University School of Divinity students and faculty. Not a few students’ vocational journeys have brought them from the mountains of Appalachia to Forsyth County to enroll in our Master of Divinity degree program. Other students have ventured to Appalachian mountain places through our courses or other programs, and their vocational identities have been shaped by their journeys.

Marcus McGill (MDiv., ‘15), now a pastor in Shelby NC, grew up in Appalachia. Conversations with Appalachia’s community leaders increased McGill’s awareness both of the challenges people in the region face and the innovative efforts communities are making to promote communal health and flourishing. Today, McGill serves a church on the fringes of the Appalachia region. What he encountered through the Appalachia course made McGill more aware of the role of congregations in solving significant societal and cultural problems: “Whether we partner with schools to ensure all children have food, advocate for senior adults to have access to proper health care services, or speak out against injustices that our communities face, we have a job to do.” (M. McGill; personal communication; March 2019.)

Not too long ago I visited Lord’s Acre,^3^ a “giving garden” just outside Asheville in Fairview NC, where several divinity students have been interns. Lord’s Acre brings people and community’s together around organic food: “What we believe in is taking peoples’ strengths, passions, talents, and creativity and weaving those into solutions on issues like food security, loneliness, prejudice, and the isolation of modern society, all of which play a part in why people are hungry. Yes, we believe a garden and the acts of learning, growing and eating food together, can accomplish all that. *We believe it because we see it every day*.”^3^ When I visited Lord’s Acre, pairs of weary work shoes were lined up at one of the garden entranceways to greet me; people who come to Lord’s Acre get their feet and hands dirty in the rich Appalachian soil of community-making.

The image of those dusty work shoes lingers with me as I think about the amazing work so many communities are doing in the North Carolina mountains. Dust tells a story. The biblical Genesis creation story depicts God forming human beings from dust—from humus, the soil of the earth. As a religious image in biblical and Christian liturgical traditions, dust is viewed as the stuff of which humans are made and the stuff to which humans return upon death. This image reminds me that human beings are in their very physicality part of the earth and with that reality comes a responsibility for care for the earth and its communities. Dust fertilizes the future, if you will. Ministry students and religious leaders have traveled North Carolina mountain pathways as part of some of our school’s programs. They have returned to our campus and journeyed on to other ministry places with the dust from their Appalachian travels clinging to their feet, minds, and hearts. I celebrate this dust and the ways, seen and unseen, that it is fertilizing the future of ministries and communities in Appalachia and beyond.

## References

[1] Bahnson F Wirzba N Making peace with the land: God’s call to reconcile with creation Westmont IL InterVarsity Press 2012

[2] Crainshaw J Teaching the sacraments through profane religious experiences; in Grounding Education in Environmental Humanities: Exploring Place-Based Pedgagogies in the South Johnston LF Aftandilian D Abingdon UK Routledge 2018

[3] The Lord’s Acre Learn, Grow, Share! thelordsacre.org/

